# *Primula maershanensis* (Primulaceae), a new species in *Primula* sect. *Auganthus* from Sichuan, China

**DOI:** 10.3897/phytokeys.274.191386

**Published:** 2026-04-28

**Authors:** Ming-yun Sheng, Yuan-mi Wu, Jiu-lin Gu, Hong-qiang Lin, Wei Zhou, Lei Zheng, Zhi-kun Wu

**Affiliations:** 1 Department of Pharmacy, Guizhou University of Traditional Chinese Medicine, Guiyang, 550025, Guizhou, China Kunming Institute of Botany, Chinese Academy of Sciences Kunming China; 2 Germplasm Bank of Wild Species & Yunnan Key Laboratory of Crop Wild Relatives Omics, Kunming Institute of Botany, Chinese Academy of Sciences, Kunming, 650201, Yunnan, China Guizhou University of Traditional Chinese Medicine Guiyang China; 3 School of Pharmacy, Sichuan College of Traditional Chinese Medicine, Mianyang, 621000, Sichuan, China Sichuan College of Traditional Chinese Medicine Mianyang China; 4 Sichuan Wolong National Natural Reserve Administration Bureau, Wenchuan, 623006, Sichuan, China Sichuan Wolong National Natural Reserve Administration Bureau Wenchuan China

**Keywords:** Conservation status, diversity, maer shan bao chun, nomenclature, Sichuan, taxonomy

## Abstract

*Primula maershanensis* J.L.Gu & Z.K.Wu, a new species of Primulaceae from Sichuan, China, is described and illustrated. Morphological evidence supports the placement of *P. maershanensis* within *Primula* sect. *Auganthus*, a section characterized by leaves that are shallowly to deeply lobed and covered with hairs and by a distinctively broad calyx with a flattened base. The new species is distinguished from other species in this section by its short rhizome and nearly fleshy roots; subpurple petioles and scapes; leaf blade suborbicular, wider than long, margin palmately 5–9-lobed to the middle of the blade; and a bright yellow corolla associated with long-homostylous flowers. Information on the distribution, morphological comparisons with closely related species, and the conservation status of the new species is also provided, along with a key to the known species of *Primula* sect. *Auganthus*.

## Introduction

*Primula* L. is one of the largest genera within the family Primulaceae, comprising up to 549 species worldwide (POWO 2026). It exhibits a predominantly Holarctic distribution pattern, with the majority of species occurring in temperate and alpine zones of the Northern Hemisphere, while only a limited number are found in the Southern Hemisphere (Hu 1990, 1994; Richards 2002). The southwestern region of China, particularly the Himalayan–Hengduan Mountains, serves as the primary diversity center for the genus, harboring more than 300 species that are mainly concentrated in western Sichuan, eastern Xizang, and northwestern Yunnan (Hu 1990; Hu and Kelso 1996; Richards 2002; Yang 2023). The taxonomy of this genus remains challenging due to the combined effects of hybridization, evolutionary transitions between heterostylous and homostylous floral systems, and adaptive radiation (Mast et al. 2001; Ren et al. 2015; He et al. 2021; Zhang et al. 2023).

*Primula* sect. *Auganthus* (Link) Pax ex Balf. f. (39: 139, 1913) (Balfour 1913) represents a small lineage within the genus *Primula*. Traditionally, this section comprised only two or three species: *Primula filchnerae* R.Knuth, *Primula sinensis* Sabine ex Lindley, and *Primula rupestris* Balf. f. & Farrer (Hu 1990; Halda 1992; Hu and Kelso 1996; Hao et al. 2002; Xu et al. 2020). Between 2023 and the present, four new species have been described, namely *Primula xingshanensis* Y.B.Wang, *Primula jiangyouensis* J.L.Gu & Z.K.Wu, *Primula rongrong* S.Y.Zhang, Y.Hu & J.W.Shao, and *Primula fujiangensis* J.L.Gu, Ying F.Hu & S.Y.Zhang (Wang et al. 2023; Gu et al. 2025; Zhang et al. 2025). These additions bring the total number of accepted species in this section to seven. These species are predominantly distributed in the border regions of Hubei, Shaanxi, and Sichuan in north-central China.

As a biodiversity hotspot in China, Sichuan harbors approximately 177 species of *Primula* (Primulaceae) distributed across the region (Yang 2023). With intensified botanical exploration, eight new *Primula* species have been documented in Sichuan in the past few years (Ju et al. 2023; Li et al. 2023; Luo et al. 2023; Li et al. 2024; Gu et al. 2025; Shuai et al. 2025; Zhang et al. 2025). Currently, a total of 185 *Primula* species are recognized in Sichuan.

In January 2026, during a field survey conducted in the Guangyuan area of Sichuan Province, a region characterized by highly diverse karst vegetation, a distinctive *Primula* population with atypical morphology was discovered. The plants exhibit lobed leaves, a dense indumentum covering the entire plant, and a conspicuously inflated calyx with a broad, flattened base, characters typical of *Primula* sect. *Auganthus*. However, their floral morphology is unique, leaf blade suborbicular, wider than long, palmately 5–9-lobed to approximately 1/2 of its blade; and a bright yellow corolla associated with long-homostylous flowers. To clarify its taxonomic status, further investigations were conducted in and around the type locality, specimens were collected, and detailed morphological studies were carried out. Based on comprehensive morphological analyses and comparisons with relevant literature and congeneric species, this taxon is confirmed as a species new to science. Therefore, it is formally described here as a new species, accompanied by a detailed morphological description and illustrations.

## Materials and methods

Morphological observations, measurements, and descriptions (including habit, rhizome, indumentum, leaf, calyx, corolla, and flower coloration) of the new species were conducted based on both dried specimens and living plants collected from Jiange County, Sichuan Province. Comparative morphological analyses with closely related species were carried out through examination of specimens from major herbaria, including PE, P, E, IBSC, ANUB, BM, and KUN. A total of 38 specimens were examined (for species represented by more than 20 specimens, 20 specimens were examined; for species represented by fewer than 20 specimens, all available specimens were examined), as well as relevant literature (Smith and Fletcher 1947; Hu 1990; Halda 1992; Hu and Kelso 1996; Richards 2002; Wang et al. 2023; Gu et al. 2025; Zhang et al. 2025). Key morphological characters of *P. maershanensis* and its allied species within *P.* sect. *Auganthus*, namely *P. sinensis*, *P. rupestris*, and *P. jiangyouensis*, fifteen living plants from each of their respective type localities were measured using a vernier caliper. The conservation status of the new species was assessed following the guidelines of the IUCN Red List categories and criteria (IUCN Standards and Petitions Committee 2024). To facilitate species identification within *P.* sect. *Auganthus*, a key to the eight species based on primary morphological differences, is provided.

## Taxonomic treatment

### Primula maershanensis

Taxon classification

Plantae

EricalesPrimulaceae

J.L.Gu & Z.K.Wu sp. nov.

F3A67E91-661E-579F-9584-9BFFCD47F715

urn:lsid:ipni.org:names:77379332-1

Figs 1, 2, 3A, 4A

#### Diagnosis.

**Figure 1.**
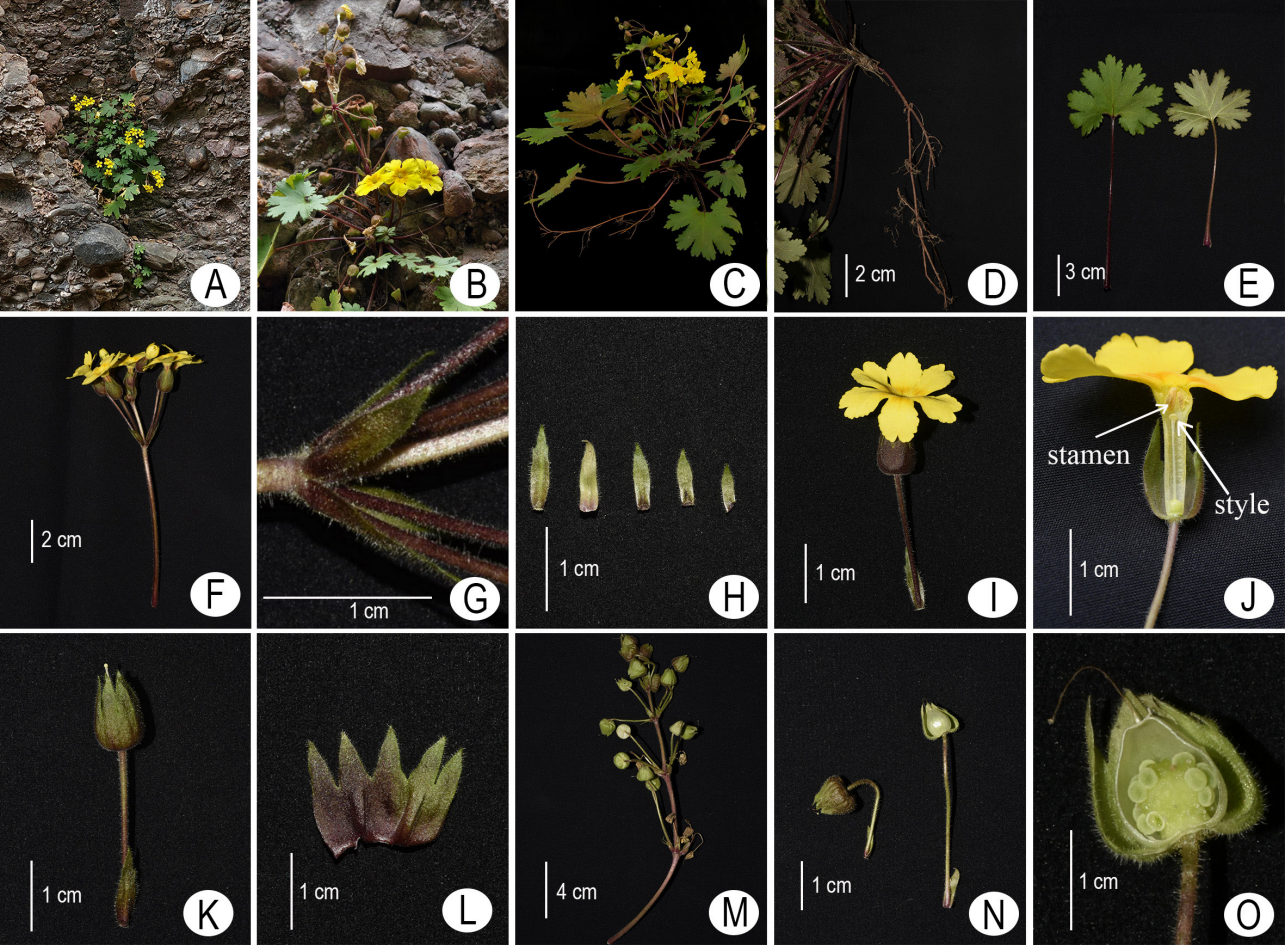
*Primula maershanensis* sp. nov. **A**. Habitat; **B, C**. Habit during flowering; **D**. Roots; **E**. Leaves, left: upper surface, right: lower surface; **F**. Flower scape; **G**. Flower bract (enlarged view); **H**. Bracts; **I**. Flower, lateral view; **J**. Dissected corolla showing anthers and stigmas; **K**. Calyx and pedicel; **L**. Outside of dissected calyx; **M**. Infructescence; **N**. Young fruits; **O**. Dissected young fruit. Photographed by Zhikun Wu, Mingyun Sheng, and Jiulin Gu.

**Figure 2.**
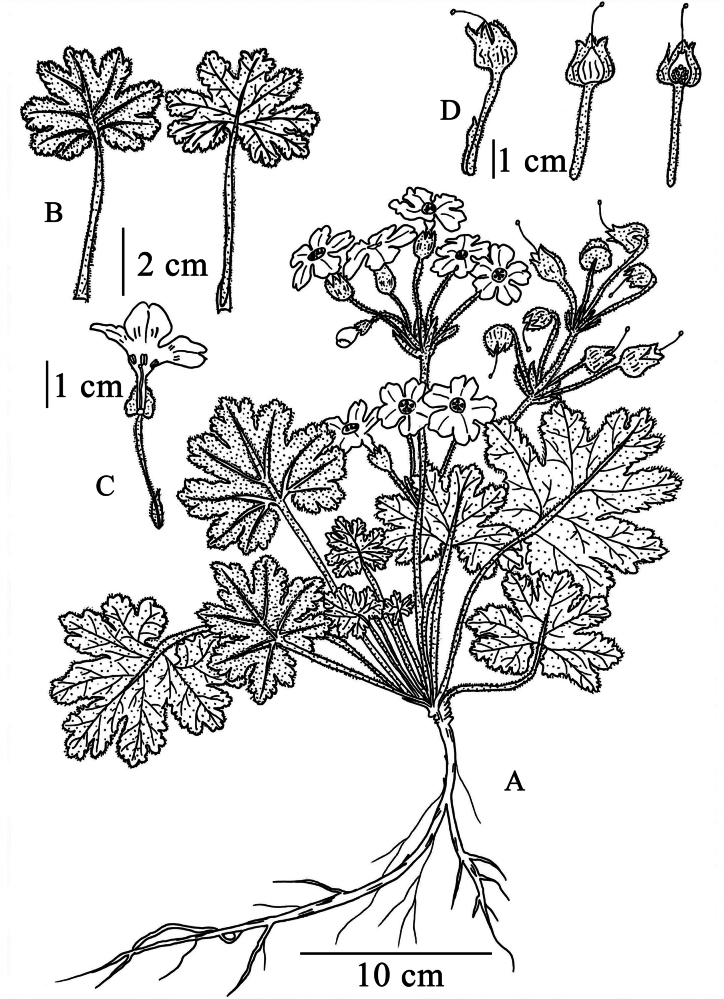
*Primula maershanensis* sp. nov. **A**. Habit; **B**. Leaves, left: lower surface, right: upper surface; **C**. Dissected corolla; **D**. Fruit and calyx, left: fruit with calyx, middle: young fruit, and right: dissected young fruit. Drawn by Ms. Mingyun Sheng.

**Figure 3.**
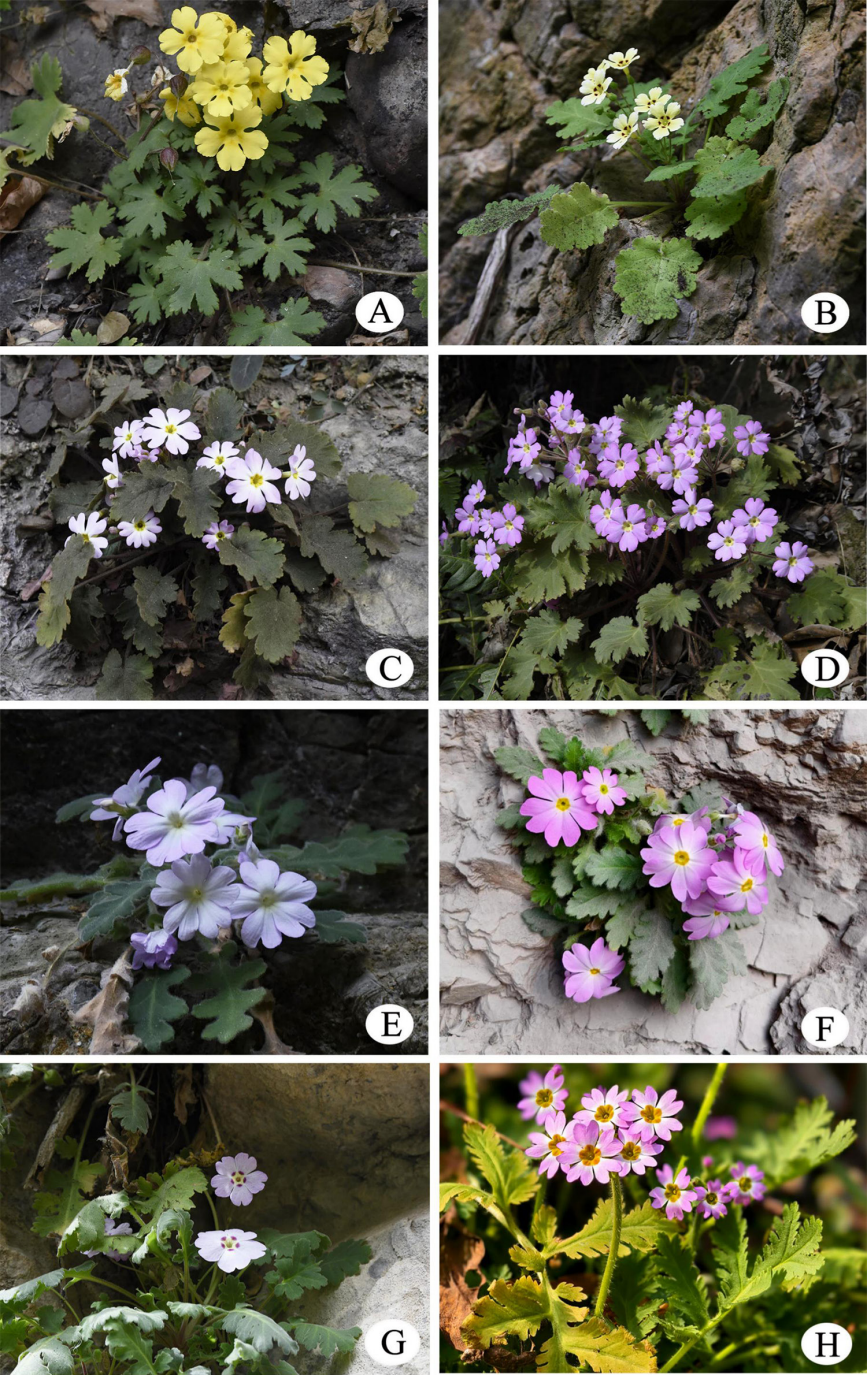
*Primula maershanensis* and seven species in *Primula* sect. *Auganthus*. **A**. *P. maershanensis*; **B**. *P. jiangyouensis* (Jiangyou County, Sichuan, type locality); **C**. *P. rupestris* (Ningqiang County, Shaanxi, type locality); **D**. *P. sinensis* (Dayi County, Sichuan); **E**. *P. rongrong* (Qingchuan County, Sichuan, type locality); **F**. *P. xingshanensis* (Xingshan County, Hubei, type locality); **G**. *P. fujiangensis* (Jiangyou County, Sichuan, type locality); **H**. *P. filchnerae* (Mian County, Shaanxi). Photographed by Zhikun Wu and Jiulin Gu.

**Figure 4.**
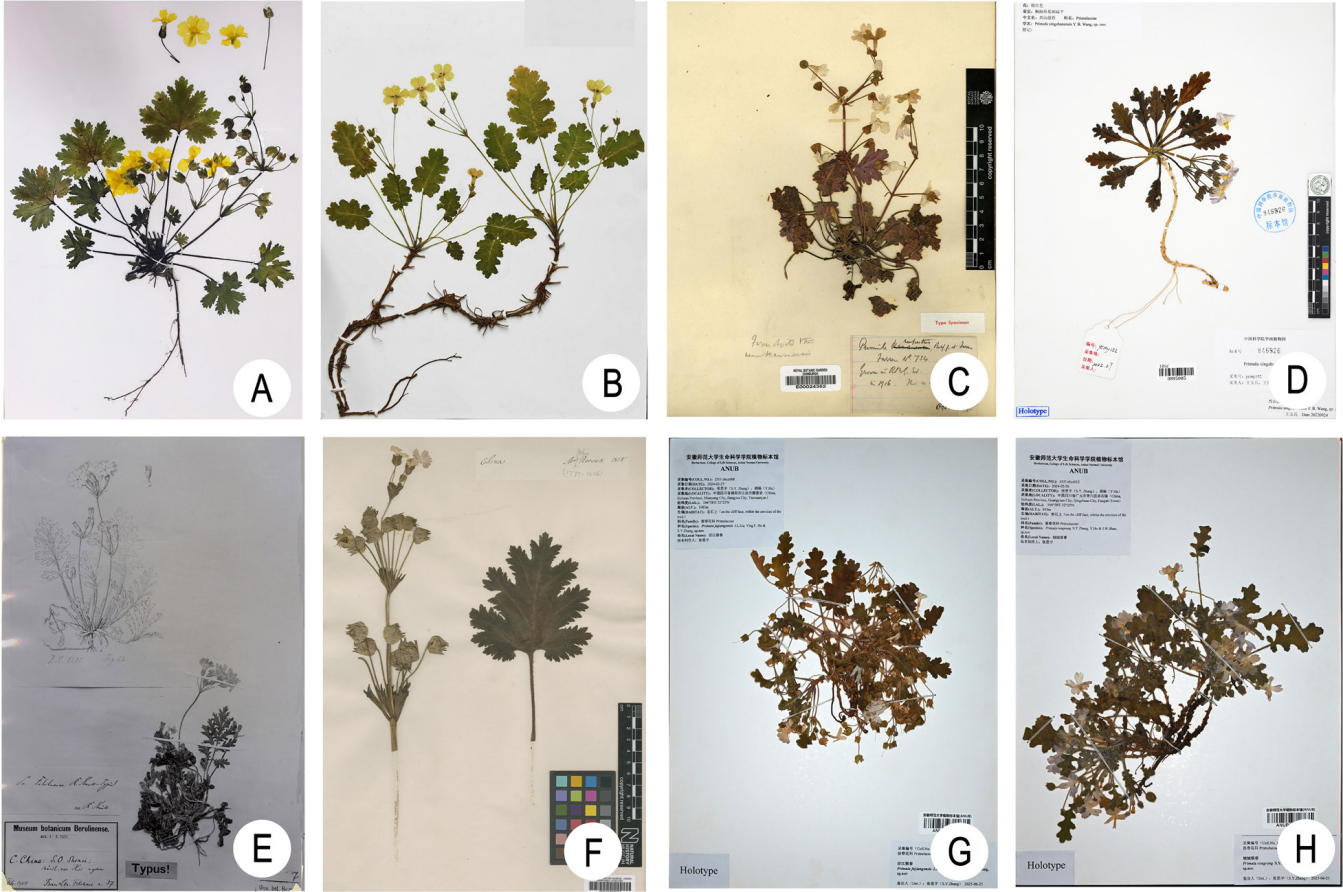
Type specimens of *Primula maershanensis* and seven species in *Primula* sect. *Auganthus*. **A**. Holotype of *P. maershanensis* (ZKWU2026010, KUN!); **B**. Holotype of *P. jiangyouensis* (GJL384, KUN!); **C**. Holotype of *P. rupestris* (Farrer 734, E!); **D**. Holotype of *P. xingshanensis* (YCMY 152, IBSC!); **E**. Type of *P. filchnerae* (photo, Filchner 37, E00024341); **F**. Lectotype of *P. sinensis* (BM barcode BM000996827); **G**. Holotype of *P. fujiangensis* (ANUB100115, ANUB!); **H**. Holotype of *P. rongrong* (ANUB100111, ANUB!).

The new species is most similar to *P. sinensis*, *P. rupestris*, and *P. jiangyouensis* in sharing leaves and stems covered with hairs, lobed leaf blades, distinctly petiolate leaves, and a distinctively broad and flat-bottomed calyx. However, it is distinguished from the latter three mainly by several morphological features: a short rhizome and nearly fleshy roots; subpurple petioles and scapes; leaf blade suborbicular, wider than long, palmately 5–9-lobed to approximately 1/2 of its blade, long-homostylous flowers with a bright yellow corolla (Figs 1, 2). For a more accurate delimitation of all known species in this section, we observed living plants and captured their photographs, in addition to examining herbarium specimens of the other seven species within this section (Figs 3, 4). The main morphological distinctions between *P. maershanensis* and *P. jiangyouensis*, *P. sinensis*, and *P. rupestris* are summarized in Table 1.

**Table 1.** Morphological comparison of *Primula maershanensis*, *P. jiangyouensis*, *P. rupestris*, and *P. sinensis*.

Characters	*P. maershanensis*	*P. jiangyouensis*	*P. rupestris*	*P. sinensis*
Habitat	Shady ledges of conglomerate cliffs	Shady ledges of limestone cliffs	Shady ledges of limestone cliffs	Shady ledges of limestone cliffs
Roots	Nearly fleshy	Fibrous and brittle	Nearly fleshy	Nearly fleshy
Rhizomes	Short, 1–2 cm long, unbranched, without dry old leaves at the apex	Stout, usually with 2–8 branches, up to 40 cm long, with numerous dry, old leaves at the apex	Comparatively stout, occasionally branched, up to 15 cm long, with numerous dry, old leaves at the apex	Comparatively stout, unbranched, up to 10 cm, without dry old leaves at the apex
Petiole	Subpurple to purplish red, 2–13 cm long	Pale red or green, 4–16 cm long	Green, 4–13 cm long	Purplish, 4–15 cm long
Leaf blade	Suborbicular, palmately 5–9-lobed to approximately 1/2 of its blade	Oblong-ovate to ovate-orbicular, pinnately 4–6-lobed to approximately 1/2 or more of the blade	Ovate-rotund to ovate-elliptic, pinnately 4–6-lobed to approximately 1/3–1/24 of its blade	Ovate to subrotund, palmately 5–9-lobed to approximately 1/2 of its blade
Corolla	Bright yellow, with a pale orange blotch at the base of lobes	Yellow, with a fan-shaped reddish-brown blotch at the base of lobes	White, pale lilac or rose, with a yellowish-green blotch at the base of lobes	White, pink, rose or purple sometimes with a rose blotch at the base of lobes
Flower	Long-homostylous	Heterostylous	Heterostylous	Heterostylous

#### Type.

China • Sichuan: Guangyuan, Jiange County, Jianmenguan town, Maer Mountain. 32°10'52"N, 105°33'24"E, 810 m alt., 18 January 2026 (fl.), *Zhikun Wu ZKWU 2026010* (holotype: KUN!; isotype: CSH!).

#### Description.

Perennial herb, with aerial parts densely covered with short glandular hairs; rhizomes short, 1–2 cm long and roots fleshy, purplish-brown to pale purple. ***Leaves*** mostly clustered at the apex of the rhizome in a rosette, including the petiole 4–20 cm long; petiole 2–13 cm long, densely covered with short glandular hairs, purplish-red; leaf blade suborbicular, 1–7 cm long, 2–8 cm wide, palmately 5–9-lobed to approximately 1/2 of its blade; lobes secondarily 3-lobed, lobules with 1–3 teeth, apex obtuse, slightly thick when fresh, papery when dry, midvein prominent, lateral veins in 2–4 pairs, adaxially slightly impressed and densely covered with short glandular hairs, abaxially raised and densely covered with glandular hairs along the veins. ***Scapes*** 6–24 cm tall, purplish-red; scapes with 1–5 whorls, each whorl with 3–10 flowers. Bracts lanceolate, entire, 8–15 mm long, apex acute, densely glandular hairy. ***Pedicel*** 1.5–5 cm long, densely glandular hairy. ***Calyx*** 7–15 mm long, base inflated and hemispherical, 5–10 mm in diameter, enlarging to 8–15 mm in diameter in fruit, mostly 5-lobed, lobes incised to one-third of its length, lobes triangular, outer surface is densely covered with short glandular hairs, while the inner surface is glabrous. ***Corolla*** bright yellow, limb 1.8–3.5 cm in diameter, throat orange-yellow, without an annular appendage; lobes obcordate, margin entire or lobed, apex bifid, with a pale orange blotch at the base. ***Flower*** long-homostylous, corolla tube 15–19 mm long, stamens inserted near the mouth of the corolla tube,13–17 mm from the base of the corolla tube; style 9–14 mm long, nearly reaching or slightly exceeding the stamens. ***Capsule*** long-conical, 5–10 mm in diameter, shorter than the calyx. ***Seeds*** globose or ovoid, approximately 1.0 mm in diameter, finely vesicular-surfaced.

#### Distribution and habitat.

**Map 1.**
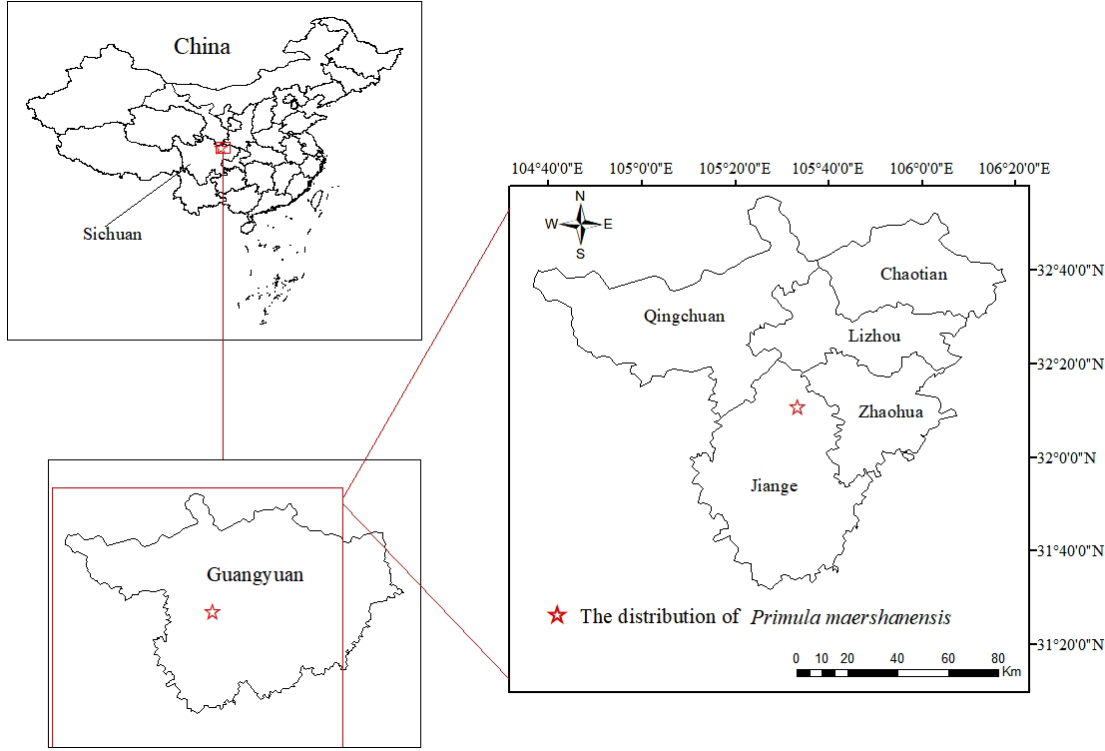
Location of the distribution of *Primula maershanensis* in Jiange, Sichuan.

The new species is currently known from the type locality near Maershan Scenic Area, Jianmenguan Town, Jiange County, Sichuan Province, China, and is primarily distributed in shady ledges of conglomerate cliffs at elevations between 800 and 1,050 meters. (Fig. 1; Map 1).

#### Phenology.

The species was observed to flower from December to February of the following year, fruiting from March to April.

#### Etymology.

The specific epithet of the new species is taken from the Chinese Pinyin "Maershan," the name of the county in northern Sichuan, China, where the type specimen was collected (Map 1).

#### Vernacular name.

Chinese Mandarin: "ma er shan bao chun“ (马耳山报春).

#### Provisional conservation status.

Critically Endangered (CR B1ab(iii)). The distribution range of this species is highly restricted, with records currently limited to its type locality in the Maershan Scenic Area. According to field observations, this species grows on shady ledges of conglomerate cliffs at elevations between 800 and 1,050 meters, and the total number of mature individuals across the population is fewer than 500. The principal threatening processes include (1) climate change-induced alteration of microclimatic conditions and (2) tourism development. Applying the IUCN Red List Categories and Criteria (Version 16.0, 2024), this assessment demonstrates that *P. maershanensis* meets the threshold for Critically Endangered (CR) status under criterion B1ab(iii), based on extent of occurrence (EOO) < 100 km^2^ (B1), severely fragmented habitat and only one location (a), with continuing decline (b), area, extent, and quality of habitat (iii).

## Discussion

*Primula* sect. *Auganthus* represents a small yet morphologically distinct assemblage within *Primula*, characterized by a broadly inflated basal portion that contrasts with the constricted upper portion that tightly adheres to the corolla tube, a feature that distinguishes it from other sections of subgenus *Auganthus*. In traditional taxonomic systems, prior to the publication of *P. xingshanensis* in 2023, this section was consistently considered to comprise only two or three species, including *P. sinensis*, *P. rupestris*, and *P. filchnerae* (Hu 1990; Halda 1992; Hu and Kelso 1996). Previously, during the identification of herbarium specimens or living plants in the field, any *Primula* specimen with a suborbicular leaf blade and an inflated calyx was typically identified as either *P. rupestris* or *P. sinensis*, without in-depth investigation into its morphological variation or the potential existence of novel species. With the intensification of field investigations over the past decade, both botanists and plant enthusiasts have discovered plants morphologically distinct from *P. sinensis* or *P. rupestris*. This has led to the recent publication of four new species: *P. xingshanensis* (Wang et al. 2023), *P. jiangyouensis* (Gu et al. 2025), *P. rongrong*, and *P. fujiangensis* (Zhang et al. 2025), based on morphological characteristics and molecular phylogenetic analyses. These discoveries have increased the number of known species in this section to seven, enriching its documented diversity. The newly discovered *P. maershanensis* possesses an inflated calyx base, indicating its placement within *P.* sect. *Auganthus*. However, its bright yellow corolla associated with long-homostylous flowers and palmately 5–9-lobed suborbicular leaf blades readily distinguish it from all other known species of *P.* sect. *Auganthus*.

In recent years, extensive field investigations on *Primula* sect. *Auganthus* have been conducted, including surveys at the type localities of these newly described species. It was found that, with the exception of *P. filchnerae*, which grows on sloping fields at forest margins, all other known species of this section inhabit shady limestone cliffs in karst areas. In contrast, the newly discovered *P. maershanensis* grows on shady conglomerate cliffs. The inherently fragmented nature of cliffs in karst regions readily leads to habitat fragmentation for species of this section. This fragmentation frequently results in species differentiation due to habitat isolation, ultimately leading to the formation of new taxa, particularly narrowly distributed species with extremely small populations. Recent investigations indicate that the newly described *P. xingshanensis*, *P. jiangyouensis*, *P. rongrong*, and *P. fujiangensis* each have very narrow distribution ranges and small population sizes, with fewer than five populations currently known for each. Consequently, species of *P.* sect. *Auganthus* serve as an excellent model group for studying species differentiation driven by habitat fragmentation in karst landscapes. The discovery of *P. maershanensis* not only enriches the species diversity of *P.* sect. *Auganthus* but also provides valuable material for investigating species differentiation in karst regions.

### Key to the species of *Primula* section *Auganthus*

From the distribution of eight species, all are endemic to China. To facilitate the identification of these eight species, a key is constructed as follows:

**Table:** 

1	Flowers yellow	**2**
–	Flowers white, lilac, pink, or rose	**3**
2	Flowers homostylous; with a pale orange blotch at the base; Leaf blade suborbicular, slightly wider than long, margin palmately lobed	***P. maershanensis* sp.nov**.
–	Flowers heterostylous; with a fan-shaped reddish-brown blotch at the base of flower lobes; leaf blade oblong-ovate to ovate-orbicular, longer than wide, margin pinnately lobed	***P. jiangyouensis***
3	Leaves oblong, length much greater than width	**4**
–	Leaves ovate to subrotund, slightly longer than wide	**7**
4	Leaves pinnately lobed, lobing extending to the midvein	***P. filchnerae***
–	Leaves pinnately or palmately lobed, lobing not reaching the midvein	**5**
5	Leaf base cuneate; lobes entire	***P. xingshanensis***
–	Leaf base cordate; lobes serrate	**6**
6	Whole plant villous; nectary ducts absent	***P. rongrong***
–	Whole plant hairy; nectary ducts purple	***P. fujiangensis***
7	Leaf rosette with wiry petioles of withered leaves at base; calyx 8–10 mm in fruit	***P. rupestris***
–	Leaf rosette without remains of old leaves at base; calyx 15–20 mm in fruit	***P. sinensis***

## Supplementary Material

XML Treatment for Primula maershanensis

## Additional information

### Artificial Intelligence (AI) use

The authors accept full responsibility for the content of the manuscript, including the disclosure of any use of AI.

No AI tools were used in the preparation of this manuscript.

### Author ORCIDs

M.-y. Sheng https://orcid.org/0009-0001-0310-4003

Y.-m. Wu https://orcid.org/0000-0001-5046-8902

J.-l. Gu https://orcid.org/0009-0007-7557-0445

H.-q. Lin https://orcid.org/0000-0001-8152-4911

W. Zhou https://orcid.org/0000-0001-5527-3776

L. Zheng https://orcid.org/0000-0001-7835-3963

Z.-k. Wu https://orcid.org/0000-0002-4583-7782

### Data availability

All of the data that support the findings of this study are available in the main text.
